# Embryotrophic effect of exogenous protein contained adipose-derived stem cell extracellular vesicles

**DOI:** 10.1186/s40104-024-01106-4

**Published:** 2024-11-03

**Authors:** Seonggyu Bang, Ahmad Yar Qamar, Sung Ho Yun, Na-Yeon Gu, Heyyoung Kim, Ayeong Han, Heejae Kang, Hye Sun Park, Seung II Kim, Islam M. Saadeldin, Sanghoon Lee, Jongki Cho

**Affiliations:** 1 College of Veterinary Medicine and Research Institute for Veterinary Science, Seoul National University, Seoul, 08826 Republic of Korea; 2 College of Veterinary Medicine, Chungnam National University, Daejeon, 34134 Republic of Korea; 3 College of Veterinary and Animal Sciences, Jhang Sub-campus of University of Veterinary and Animal Sciences, Lahore, 54000 Pakistan; 4 Korea Basic Science Institute (KBSI), Ochang, Chungcheongbuk-Do 28119 Republic of Korea; 5 Viral Disease Research Division, Animal and Plant Quarantine Agency, Gimcheon, Gyeongsangbuk-Do 39660 Republic of Korea; 6 Department of Plastic and Reconstructive Surgery, Vascularized Composite Allotransplantation (VCA) Laboratory, Johns Hopkins School of Medicine, Baltimore, MD 21205 USA; 7 Comparative Medicine Department, King Faisal Specialist Hospital & Research Centre, Riyadh, 11211 Saudi Arabia

**Keywords:** Adipose-derived stem cell, Embryonic development, Extracellular vesicles, Porcine

## Abstract

**Background:**

Extracellular vesicles (EVs) regulate cell metabolism and various biological processes by delivering specific proteins and nucleic acids to surrounding cells. We aimed to investigate the effects of the cargo contained in EVs derived from adipose-derived stem cells (ASCs) on the porcine embryonic development.

**Methods:**

ASCs were isolated from porcine adipose tissue and characterized using ASC-specific markers via flow cytometry. EVs were subsequently extracted from the conditioned media of the established ASCs. These EVs were added to the in vitro culture environment of porcine embryos to observe qualitative improvements in embryonic development. Furthermore, the proteins within the EVs were analyzed to investigate the underlying mechanisms.

**Results:**

We observed a higher blastocyst development rate and increased mitochondrial activity in early stage embryos in the ASC-EVs-supplemented group than in the controls (24.8% ± 0.8% vs. 28.6% ± 1.1%, respectively). The terminal deoxynucleotidyl transferase dUTP nick end labeling (TUNEL) assay of blastocysts also revealed significantly reduced apoptotic cells in the ASC-EVs-supplemented group. Furthermore, through proteomics, we detected the proteins in ASC-EVs and blastocysts from each treatment group. This analysis revealed a higher fraction of proteins in the ASC-EVs-supplemented group than in the controls (1,547 vs. 1,495, respectively). Gene analysis confirmed that ASC-EVs showed a high expression of tyrosine-protein kinase (*SRC*), whereas ASC-EVs supplemented blastocysts showed a higher expression of Cyclin-dependent kinase 1 (*CDK1*). SRC is postulated to activate protein kinase B (*AKT*), which inhibits the forkhead box O signaling pathway and activates *CDK1*. Subsequently, *CDK1* activation influences the cell cycle, thereby affecting in vitro embryonic development.

**Conclusion:**

ASC-EVs promote mitochondrial activity, which is crucial for the early development of blastocysts and vital in the downregulation of apoptosis. Additionally, ASC-EVs supply SRC to porcine blastocysts, thereby elongating the cell cycle.

**Supplementary Information:**

The online version contains supplementary material available at 10.1186/s40104-024-01106-4.

## Background

Stem cells (SCs) are undifferentiated cells that are an integral part of the body’s repair system because they can self-renew and differentiate into various cell types [1]. SCs can be isolated from different sources, including embryos, cord blood cells, and adults [2]. Adipose-derived SCs (ASCs) are adult SCs obtained from an individual's adipose tissue with minimal discomfort under local anesthesia [3, 4]. They are considered a readily available source of SCs because adipose tissues are largely available in individuals [3, 5].

ASCs can differentiate into several cell types, including adipocytes (fat cells), osteocytes (bone cells), chondrocytes (cartilage cells), and myocytes (muscle cells) [6]. Their highly proliferative nature and low risk of tumor formation make them useful in regenerative medicine. The regenerative potential of ASCs is believed to be due to the secretion of various biomolecules, including growth factors, cytokines, and extracellular vesicles (EVs) [7]. These substances promote tissue regeneration, modulate the immune system, and reduce inflammation [8].

EVs are membrane-bound structures secreted by almost all cells [9–11] and are crucial in cell-to-cell communication by transporting different biomolecules, including proteins, nucleic acids (messenger RNA [mRNA], microRNA, and DNA), lipids, and various metabolites [9, 12, 13]. EV-derived biomolecules delivered to target cells can affect multiple physiological processes, including cellular growth, immune responses, and tissue repair. These molecules can regulate cellular activity and different signaling pathways in target cells [14, 15]. EVs-derived RNA delivered to target cells can be translated into proteins, potentially affecting gene expression and cellular processes [16, 17]. Among all EV-derived constituents, internal proteins have garnered interest because of their diverse functionalities and potential implications in disease diagnostics and therapeutic interventions [9]. However, the functional roles of proteins and signals delivered through EVs may not always exert positive effects depending on the cell type.

Pluripotency-inducing proteins are crucial in maintaining or inducing pluripotency in cells and are mainly expressed in SCs that exhibit multilineage differentiation [18, 19]. These proteins include Octamer-binding transcription factor 4 (Oct4), sex-determining region Y [SRY]-box 2 (Sox2), Nanog, and Kruppel-like factor 4 (Klf4) [20]. SC-derived EVs contain pluripotency-inducing proteins, and these proteins can potentially alter the nature of the recipient cell when they are transferred to a recipient cell [21, 22]. This protein delivery can enhance cellular reprogramming, induce pluripotency, or modulate cellular functions in a manner similar to direct protein transfer or genetic manipulation. The presence of pluripotency-inducing proteins within SCs-EVs suggests that endoplasmic reticulum can potentially be used for several therapeutic applications, such as tissue regeneration, cell reprogramming, and disease treatment [23, 24].

Recent reports have demonstrated co-culture systems using SCs, biomaterials derived from SCs, or EVs derived from SCs with oocytes and embryos of different species, including bovine, mice, and pigs [25–28]. The in vitro production (IVP) of embryos using such co-culture systems positively affects oocyte maturation and embryonic development. Ling et al. [29] reported higher oocyte maturation rates using a conditioned medium from bone marrow-derived mesenchymal SCs. Similarly, embryo culture media supplemented with 10% bioactive material derived from embryonic SCs [26] or ASCs [27] improved the quantity and quality of IVP embryos. In bovines, supplementing in vitro culture (IVC) medium with EVs resulted in improved blastocyst quality [30, 31]. ASC-EVs have several beneficial effects on mammalian embryo development. Previous studies have shown that embryos exposed to ASC-EVs have improved embryo quality due to increased cell number, rate of blastocyst formation, and hatchability. ASC-EVs protect embryos against oxidative stress (OS), which can damage the DNA and impair embryonic development. They possess anti-inflammatory properties and can reduce uterine inflammation, which may promote embryonic implantation and development.

The development of the early embryo is a major transition period that leads to blastocyst formation and begins with rapid, nearly simultaneous mitosis [32]. These initial divisions proceed faster than adult mitosis and drive mitotic cell cycle progression. Based on these outcomes, we hypothesized that the presence of ASC-EVs in the culture medium could enhance the developmental competence of IVP embryos. Therefore, we aimed to investigate the effects of ASC-EVs supplemented medium on porcine embryonic development. Moreover, proteomic analysis of ASC-EVs was performed to examine the impact of EV influx on porcine embryo development and discern the actual functional proteins transmitted and their influence on embryo development.

## Materials and methods

### Chemicals

All chemicals and reagents not otherwise specified were purchased from Sigma-Aldrich (St. Louis, MO, USA).

### Preparation of ASC-EVs

#### Primary cell culture of ASCs

Primary cell cultures were isolated from adipose tissue as described by Zuk et al. [33]. Porcine adipose tissue was collected from a nearby slaughterhouse, stored at 4 °C, and brought to the laboratory within 1 h. The adipose tissue was finely minced using sterile surgical scissors after washing with phosphate-buffered saline (PBS) containing 75 mg/mL penicillin and 50 mg/mL streptomycin. The minced adipose tissue was transferred to a tube containing 0.1% collagenase-PBS and was then incubated at 38 °C for over 4 h to digest. The digested tissue was sequentially filtered through 100-μm and 70-μm cell strainers (SPL, Korea). The filtered lysates were centrifuged at 400 × *g* for 5 min, and the supernatant was removed. The pellet was then washed three times with PBS and treated with 160 mmol/L ammonium chloride at room temperature for 5 min to remove red blood cells. It was then cultured in Dulbecco’s Modified Eagle Medium (DMEM) supplemented with 10% fetal bovine serum under 5% carbon dioxide (CO_2_) at 37 °C. The cells were cultured in a new medium the following day after confirming their attachment and then cultured for approximately 7 d.

#### ASCs marker detection and differentiation

ASCs were characterized by detecting the target markers CD29, CD34, CD44, and CD90 using a BD Accuri™ C6 plus (Becton Dickinson, BD Biosciences, Franklin Lakes, NJ, USA) for mesenchymal component analysis [34, 35]. In brief, cultured ASCs were used when they reached 80% confluence after passage 3. Cells were harvested using 0.25% trypsin–EDTA, washed with PBS, and then incubated with antibodies against MSC-specific markers (Table S1). The concentration of each antibody was used according to the manufacturer's recommendations. After incubating for 30 min in the dark, primary antibodies that were not conjugated with fluorophores were subjected to centrifugation, followed by incubation with secondary antibodies for an additional 30 min. The cells were then centrifuged at 500 × *g* for 5 min, resuspended in PBS, and analyzed using flow cytometry. The differentiation ability and target markers of the mesenchymal SCs were also examined to characterize the isolated ASCs. ASC differentiation was induced using the method outlined in the 2020 paper by Robert et al. [36], following adipogenesis, osteogenesis, and chondrogenesis protocols (Table S1) [36–38].

#### Establishment of ASC-EVs

We followed the EVs establishment methods used in our previous study [39]. In brief, the established ASC-EVs were cultured in serum-free medium to obtain the conditioned medium, from which apoptotic bodies and cellular debris were removed through centrifugation. Subsequently, the ASC-EV pellets were obtained via ultracentrifugation. The obtained ASC-EV pellets were resuspended in PBS, and the size distribution and concentration of ASC-EVs were measured using Nanoparticle Tracking Analysis (NTA, Nanosight NS300, Malvern Instruments, Worcestershire, UK). Finally, the isolated ASC-EVs were visualized using Cryo-TEM, as in our previous studies also others [40–43]. For sample preparation, glow-discharged Quantifoil R1.2/1.3 Cu 300 grids (Quantifoil, Jena, Germany) and a Vitrobot Mark IV (Thermo Fisher Scientific, WA, USA) were utilized. Ultimately, imaging of ASC-EVs was performed using a Glacios microscope (Thermo Fisher Scientific). Protein from ASC-EVs was extracted and mixed with 4× Laemmli sample buffer (Bio-Rad Laboratories, Hercules, CA, USA). The samples were boiled, loaded onto a 12% polyacrylamide gel, and subjected to electrophoresis. Protein was transferred to a PVDF membrane, blocked, and probed overnight with primary antibodies against β-Actin, CD9, CD81, and CD63 (mouse monoclonal IgG1 κ β-actin antibody, mouse monoclonal IgG1 κ CD9 antibody, mouse monoclonal IgG2b κ CD81 antibody (Santa Cruz Biotechnology Co., Ltd., Dallas, TX, USA), and recombinant Anti-CD63 antibody (Abcam, Cambridge, MA, USA). After washing, the membrane was incubated with a horseradish peroxidase-conjugated secondary antibody. Subsequently, the membrane was incubated with SmartGene ECL High Femto Solution (SmartGene, Daejeon, Korea) to detect chemiluminescence signals using the iBright 750 imaging system (Invitrogen, Marsiling Industrial Estate, Singapore) [39].

#### Liquid chromatography–Tandem mass spectrometry analysis

The process was performed using the method from our previous study [39, 44]. An LTQ-Velos ESI ion trap mass spectrometer (Thermo Fisher Scientific) was used to obtain the mass spectrometry and Tandem mass spectrometry (MS/MS) spectra. MASCOT 2.4 was employed for the analysis of MS/MS data, with a false discovery rate set at 1%. Protein concentrations were calculated using the exponentially modified protein abundance index and represented as mol%. Each experiment was conducted with three technical replicates.

### Effects of ASC-EVs on porcine embryonic development

#### Preparation of in vitro porcine embryo

Parthenogenetic embryos were produced following our laboratory’s methods [45, 46]. Porcine ovaries were sourced from a nearby slaughterhouse, transported in a saline solution (0.9%) containing antibiotics at 34–36 °C. Cumulus oocyte complexes (COCs) from 3–8 mm antral follicles were aspirated and the follicular fluid was settled. High-quality COCs were chosen and washed thrice in a TLH medium with 0.05% polyvinyl alcohol. The selected COCs underwent IVM in TCM-199 medium with supplements for 22 h, followed by another 22 h in hormone-free medium. Parthenogenetic embryos were created by removing cumulus cells from matured COCs, treating oocytes with an activation solution, and applying an electric pulse. The embryos were cultured in PZM-5 medium with ASC-EVs at a concentration of 1.5 × 10^7^ particles/mL under controlled atmospheric conditions.

#### Detection of reactive oxygen species and intracellular glutathione

Reactive oxygen species (ROS) and glutathione (GSH) levels were measured as described previously [47]. To measure ROS levels, 2′,7′-dichlorodihydrofluorescein diacetate (H2DCFDA) was used, while intracellular GSH levels were determined using Cell Tracker™ Blue with the CMF2HC dye. Approximately 10−13 embryos from each group (control and ASC-EVs) were incubated in TLH-PVA with 10 µmol/L H2DCFDA and 10 µmol/L CMF2HC for 30 min in the dark. The embryos were washed with Dulbecco’s PBS containing 0.1% PVA. ROS and GSH levels were then analyzed using an epifluorescence microscope (Leica DM IRB, Wetzlar, Germany) with filters set at 460 nm for ROS and 370 nm for GSH. Fluorescence intensities were captured and saved as TIFF files, and band densities were quantified using Fiji software.

#### Mitochondrial activity in the early embryo stage

Embryos at the 2- to 4-cell stage were subjected to three washes with PBS/PVA, followed by incubation at 37 °C for 30 min in 500 nmol/L MitoTracker Red CMXRos. Post-staining, the embryos underwent three 10-min washes in PBS/PVA. Each embryo was fixed in 3.7% paraformaldehyde at room temperature for 30 min, followed by three 10-min washes in PBS/PVA. Subsequently, the nuclei were stained with DAPI for 10 min at room temperature, mounted on slides, and observed using an epifluorescence microscope (Leica DM IRB, Wetzlar, Germany) [45].

#### Terminal deoxynucleotidyl transferase dUTP nick end labeling assay

To evaluate cellular apoptosis in porcine blastocysts, a TUNEL assay was conducted using an In Situ Cell Death Detection kit from Roche. Blastocysts at 7 d of age were fixed in 4% paraformaldehyde and washed with dPBS containing PVA (1%). For membrane permeabilization, blastocysts were exposed to 1% Triton X-100 solution in dPBS for 1 h at room temperature. After additional washes in PVA-supplemented dPBS, blastocysts were stained with fluorescein-conjugated deoxyuridine triphosphate and terminal deoxynucleotidyl transferase at 38.5 °C for 1 h. Post-staining, blastocysts were washed in PVA-supplemented dPBS and mounted on glass slides using VECTASHIELD® Antifade Mounting Medium with DAPI. Subsequently, the number of apoptotic cells and total nuclei were observed using an inverted fluorescence microscope MF52-N (Mshot, Guangzhou, China) [39, 44, 45].

#### Quantitative real-time polymerase chain reaction

The expression levels of genes related to cell reprogramming and embryonic competence were assessed using quantitative polymerase chain reaction (qPCR). Total RNA was extracted from 7 d blastocysts using the RNeasy Micro Kit (QIAGEN, Hilden, Germany) as per the manufacturer's instructions. Complementary DNA (cDNA) was synthesized with 2× RT Pre-Mix (BioFACT, Daejeon, Korea). The expression levels of target genes were quantified using relative quantitative real-time PCR with a CFX96 real-time PCR detection system (Bio-Rad, Singapore) and SYBR 2× Real-Time PCR Pre-Mix (BioFACT, Daejeon, Korea) [44]. The relative mRNA expression levels of each target gene were normalized to that of the internal control gene Glyceraldehyde 3-phosphate dehydrogenase and quantified (Table S2).

### Proteomics analysis of ASC-EVs and investigation of ASC-EV mediated SRC influx effects

#### Protein conversion

The UniProt database (https://www.uniprot.org/) was used to check the equivalence of protein IDs in the form of official gene names. Official gene names were analyzed, replicate gene names were filtered out, and the final gene sets were processed for functional analysis.

#### Functional analysis

The Database for Annotation, Visualization, and Integrated Discovery (DAVID) (https://david.ncifcrf.gov/) was used for the functional analysis of genes. Gene Ontology (GO) terms, including biological process (BP), cellular component (CC), and molecular function (MF), were analyzed. Kyoto Encyclopedia of Genes and Genomes (KEGG) pathway analysis was performed using the KEGG database (https://www.genome.jp/kegg/) incorporated into DAVID. The GO terms were arranged based on their significance (*P*-values), and the top 15 GO terms were included in this analysis in addition to the top five pathways. All results were visualized as bar plots generated using R programming language (https://www.r-project.org/) and bioinformatics tools (https://www.bioinformatics.com.cn/).

#### Network analysis

Cytoscape software (version 3.6.1; https://cytoscape.org/) was used to generate a protein–protein interaction network utilizing the STRING plugin, with a confidence score of 0.4. The ClueGO plugin (version 2.5.9) was used in Cytoscape to visualize the network based on GO, including BP, CC, MF, and pathways. The CytoHubba plugin (version 1.5) was used to generate highly connected hub genes based on their degree of connectivity.

#### Supplementation of ASC-EVs-derived SRC in porcine embryo development

SRC, a non-receptor tyrosine kinase, actively regulates cellular proliferative responses [48]. We treated preimplantation porcine embryos with an SRC inhibitor to determine the effect of the SRC protein found in ASC-EVs on them. To investigate the effect of SRC through ASC-EVs, we performed experiments in four groups: the control group (control), the SRC inhibitor group (SRC Inh), the group supplemented with SRC inhibitor and ASC-EVs (SRC inh + ASC-EVs), and the group supplemented with ASC-EVs only. According to Yiming Wang’s research in 2016 [49], we treated the embryos with 10 μmol/L of SRC inhibitor for 30 min before adding ASCs-EVs to the culture. We then examined the changes in the SRC protein and AKT pathway through Western blotting in embryos at the 2- to 4-cell stage and investigated their effect on the blastocyst production rate.

#### Western blotting

To investigate the impact of SRC delivered to porcine embryos through ASCs-EVs supplementation, Western blot analysis was conducted on 2- to 4-cell stage embryos across various experimental groups including control, SRC Inh, SRC inh + ASC-EVs, and ASC-EVs only. Embryos at the 2- to 4-cell stage were pooled over a week to form a single experimental sample. Antibodies utilized in this study are detailed in Table S3. Protein extraction from 2- to 4-cell stage embryos in each group was performed using PRO-PREP™ Protein Extraction Solution (iNtRON Biotechnology, South Korea). Following manufacturer's instructions, 20 µg protein samples from each group were mixed with 4× Laemmli sample buffer (Bio-Rad Laboratories, Hercules, CA, USA) and boiled at 95 °C for 5 min before loading onto a 12% polyacrylamide gel. Electrophoresis was conducted at room temperature using a constant voltage of 100 V for 2 h, and separated proteins were transferred to a PVDF membrane. Following transfer, PVDF membranes were blocked with 5% skim milk solution and incubated overnight with primary antibodies at 4 °C. After washing, membranes were incubated with secondary antibodies, followed by incubation with SmartGene ECL High Femto Solution (SmartGene, Daejeon, Korea). Chemiluminescence signals were detected using an iBright 750 imaging system (Invitrogen, Marsiling Industrial Estate, Singapore). Band densities were quantified using Fiji software.

### Experimental design

Our experiment was conducted in 3 parts.

Exp. 1 (Preparation of ASC-EVs): The first experiment involved culturing ASCs and establishing a cell line, followed by the isolation of ASC-EVs using a conditioned medium obtained from ASCs. The concentration and size distribution of the isolated ASC-EVs were analyzed using NTA and validated by visualizing the EVs using Cryo TEM.

Exp. 2 (Investigation of embryotrophic effects): ASC-EVs were supplemented during porcine embryo culture to investigate their effects on embryonic development. We experimented with a control group and an experimental group supplemented with ASC-EVs in embryos. ASC-EVs were treated at a concentration of 1.5 × 10^9^ particles/mL, and the blastocyst formation rate of cultured embryos was compared. Additionally, to compare the quality of early embryos, GSH, ROS levels, and mitochondrial activity were investigated in embryos at the 2- to 4-cell stage. Finally, the effect of ASC-EVs addition on the cell apoptosis rate and changes in gene expression levels within the blastocyst were investigated.

Exp. 3 (Investigation of protein content in ASC-EVs): In the second experiment, ASC-EVs had a positive effect on porcine embryonic development. However, it was difficult to precisely determine which molecules within the EVs influenced the embryos. Therefore, we conducted a proteomic analysis to identify the proteins present in ASC-EVs. Notably, many proteins were detected, among which SRC was highly abundant. Consequently, we created 4 groups (control, SRC Inh, SRC Inh + ASC-EVs, and ASC-EVs) to investigate the effects of ASC-EV-derived SRC influx on porcine embryos.

### Statistical analysis

Data analysis was conducted using SPSS version 22 (IBM, USA). Results were expressed as mean ± standard error of the mean (SEM), with statistical significance defined as *P*-value < 0.05. Analysis was performed utilizing a generalized linear model and one-way analysis of variance (ANOVA), with Tukey’s multiple comparison test applied for post-hoc analysis.

## Results

### Characterization of ASCs

**Fig. 1:**
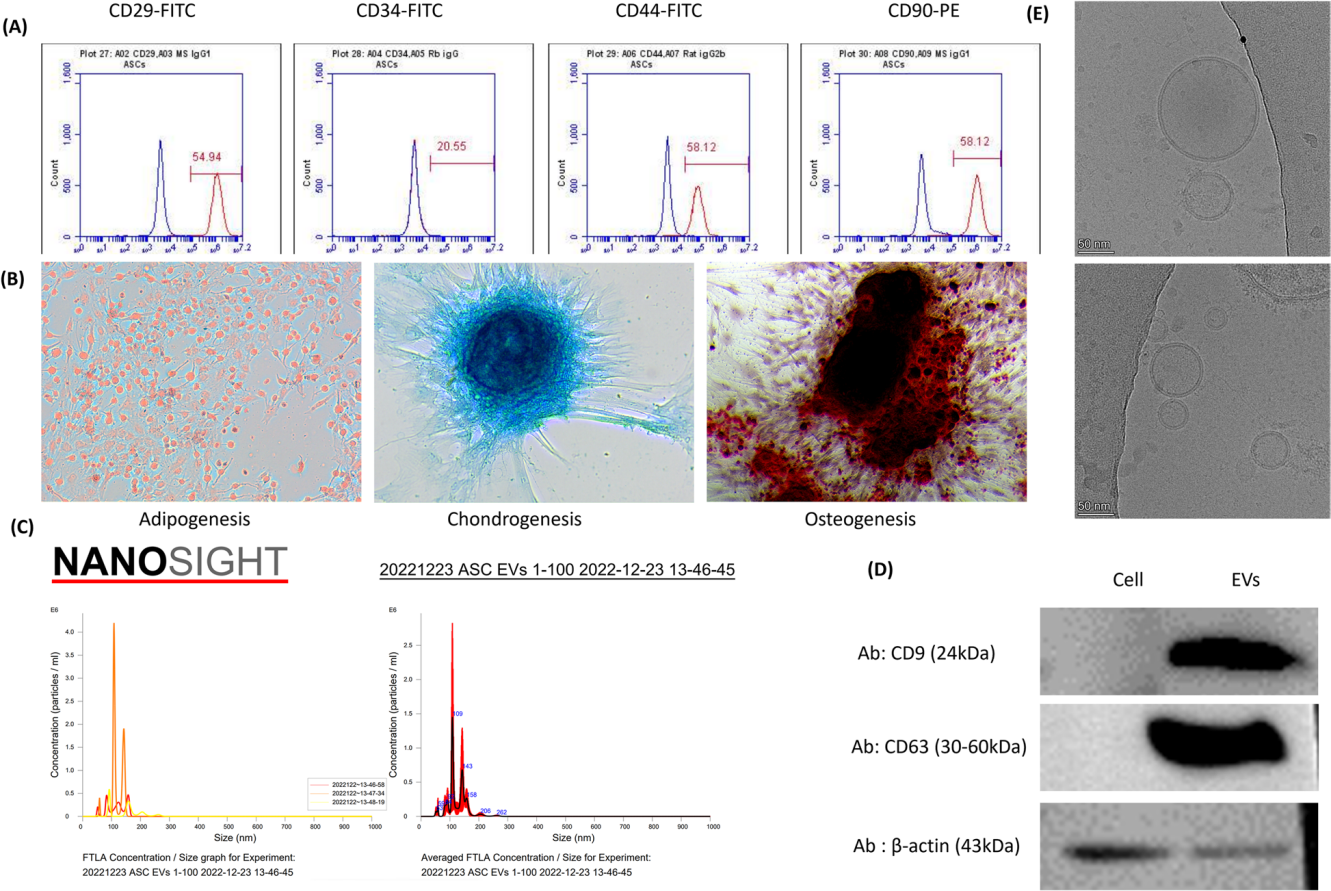
ASC characterization. To immunotype, the markers of ASC were analyzed with CD29, CD34, CD90 as positive and CD31, CD44, CD105 as negative. To identify multilineage differentiation, ASC were differentiated into (**B**) adipocyte, chondrocyte and osteocyte. Differentiated cells were stained each specific dye (Oil Red O, Alcian Blue, Alizarin Red). Morphology and size profiles of extracellular vesicles (EVs) derived from ASCs. **C** Size distribution plot, as per nanoparticle tracking analysis. **D** Western blot for the characterization of isolated ASC-EVs. **E** Visualization of ASC-EVs using by cryo-TEM

Flow cytometry was used to detect positive stem cell markers CD29, CD44, and CD90, as well as the negative marker CD34 in isolated ASCs. To confirm the differentiation potential of SCs, ASCs were induced to differentiate into adipocytes, chondrocytes, and osteocytes, and staining was confirmed using Oil Red O, Alcian Blue, and Alizarin Red (Fig. 1A and B).

### Characterization of EVs derived from ASCs

EVs derived from ASCs were visualized and characterized as described previously [39, 40]. Cryo-TEM and NTA were used to visualize and characterize ASC-EVs. Cryo-TEM was used to observe the membrane structure and size of the ASC-EVs morphologically, and the size distribution of EVs isolated using NTA was confirmed. NTA results showed that ASC-EVs with an average length of 129.7 ± 10.6 nm were isolated (Fig. 1C). The morphology of the isolated ASC-EVs was also visualized using cryo-EM (Fig. 1D).

### Preimplantation development of parthenogenetic embryos with ASC-EVs

The development of individual embryos after ASC-EV supplementation was compared by cultivating 712 embryos, with 352 and 360 embryos in the control and ASC-EV-supplemented groups, respectively. The different groups showed no significant differences in the cleavage rate of the embryos. Conversely, the blastocyst formation rate was significantly increased in the ASC-EVs group (24.8% ± 0.8% vs. 28.6% ± 1.1%, for control and ASC-EVs, respectively, Table 1).

**Table 1:** Effect of ASC-derived EVs in preimplantation porcine parthenogenetic embryo development

Groups	Number of embryos		
	**Cultured**	**Cleaved (%)**	**Developed from Bl. (%)**
Control	352	250 (70.9 ± 2.6)	87 (24.8 ± 0.8)^a^
ASC-EVs	360	262 (72.8 ± 1.7)	102 (28.6 ± 1.1)^b^

^a,b^Within a column, values with different superscript letters vary significantly among the four groups (*P* < 0.05, *n* = 7)

*ASC-EVs *Extracellular vesicles derived from adipose-derived stem cells, *Bl. *Blastocyst

### Effects of ASC-EVs supplementation on embryo quality during the 2- to 4-cell stage

**Fig. 2:**
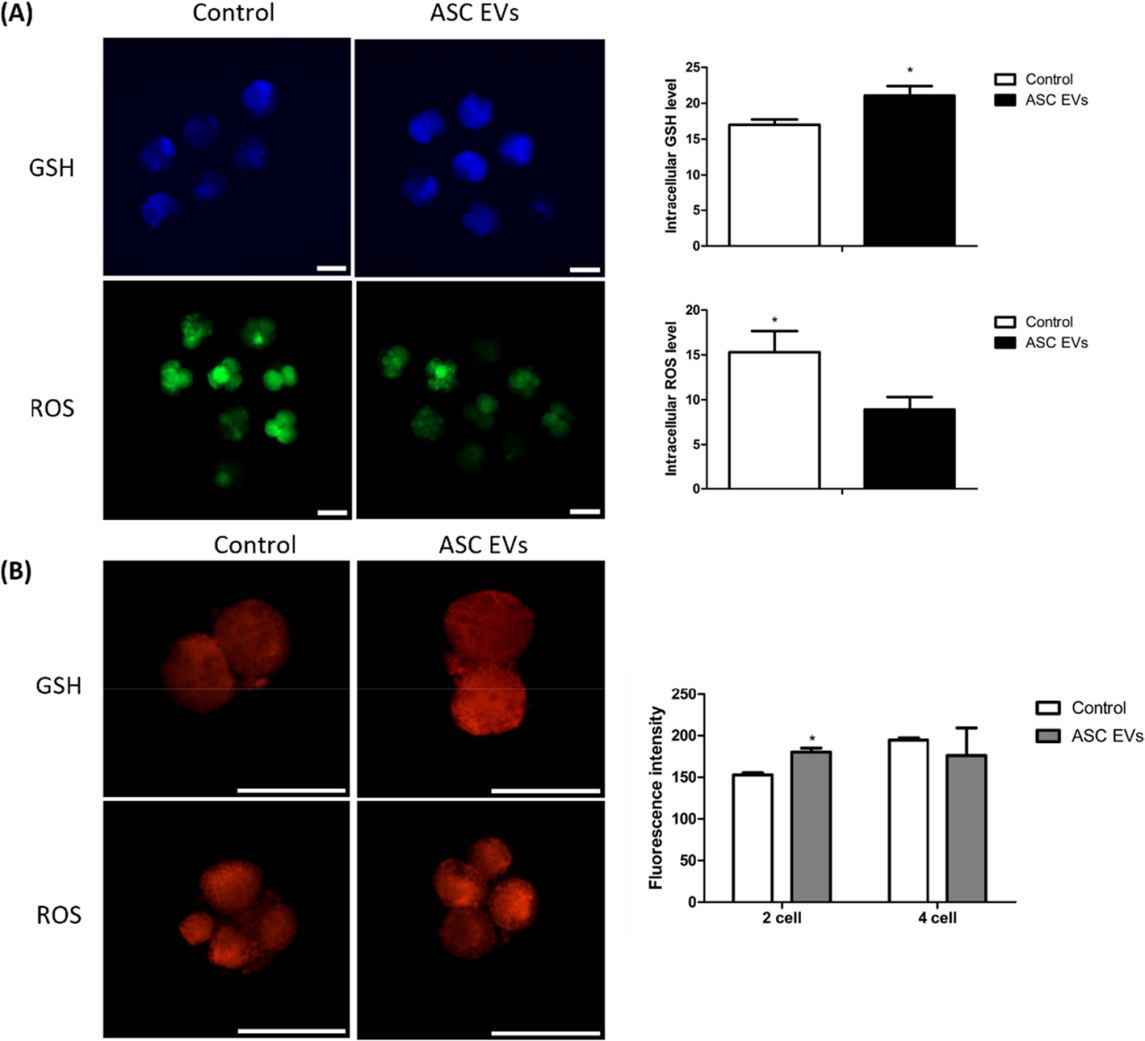
Qualitative assessment of early-stage embryos. **A** Expression analysis of inter intracellular glutathione and reactive oxygen species on 2- to 4-cell stage embryo through fluorescence staining. **B** The 2- to 4-cell stage embryos stained with Mito Tracker Red and visualized using the confocal microscope. The scale bar represents 100 μm. * means a significant difference at *P* < 0.05

Intracellular GSH and ROS levels were determined to assess the effects of ASC-EV supplementation on embryonic development. The results demonstrated that intracellular GSH levels were significantly elevated in the ASC-EV-supplemented group compared with the control group. However, ASC-EV supplementation significantly reduced ROS levels, indicating reduced OS during early embryonic development (Fig. 2A).

Moreover, mitochondrial activity was detected in the 2- to 4-cell stage embryos using the Mito Tracker Red stain. According to the fluorescence value, mitochondrial activity during the 2-cell stages was significantly increased in the ASC-EV-supplemented group. Notably, a significant difference was observed in the 2-cell stage. In contrast, in the 4-cell stage embryos, there was a tendency for mitochondrial activity to decrease in the ASC-EV group; however, no significant difference was observed. Supplementation with ASC-EVs resulted in significant changes in mitochondrial activity during the early onset of embryonic development (Fig. 2B).

### Assessment of apoptosis in blastocysts

**Fig. 3:**
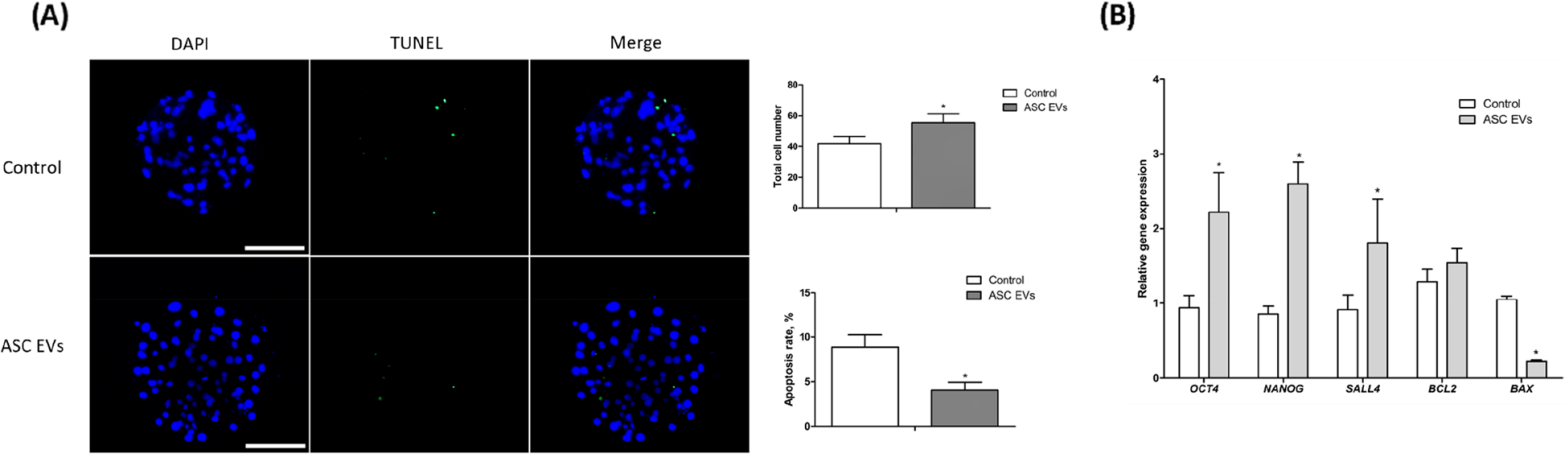
Qualitative assessment of porcine blastocysts. **A** Assessment of apoptosis in porcine parthenogenetic embryos, and comparison of total cell count. **B** Relative quantitative analysis of mRNA transcripts expression by real-time PCR. Control samples were set as arbitrary units, and the target genes were expressed as fold-change of the corresponding control relative to the housekeeping gene (*GAPDH*). The scale bar represents 100 μm. * means a significant difference at *P* < 0.05. mRNA, messenger RNA; PCR, polymerase chain reaction; GAPDH, Glyceraldehyde 3-phosphate dehydrogenase

The number of apoptotic cells was measured in developing blastocysts to confirm the positive effects of ASC-EV supplementation on embryonic development. The TUNEL assay results demonstrated a significant increase in the total number of cells in the blastocysts derived from the ASC-EV-supplemented group compared with those from the control group. Therefore, supplementation with ASC-EVs significantly reduced the number of apoptotic cells (Fig. 3A).

### Gene expression

A qPCR was performed to determine the effects of ASC-EV supplementation on the expression of cell death- and pluripotency-related genes in blastocysts. The results showed that the expression levels of pluripotency-related genes, including *OCT4*, *NANOG*, and *SALL4*, were significantly higher in blastocysts produced in the ASC-EV-supplemented group than those produced in the controls. Additionally, the expression level of the proapoptotic gene *BAX* was significantly reduced in the ASC-EV-supplemented group compared with the control group. However, the expression level of the anti-apoptotic gene *BCL2* did not show any significant difference between both groups (Fig. 3B).

### Protein contents of ASC-EVs

#### Detection of protein and functional analysis

**Fig. 4:**
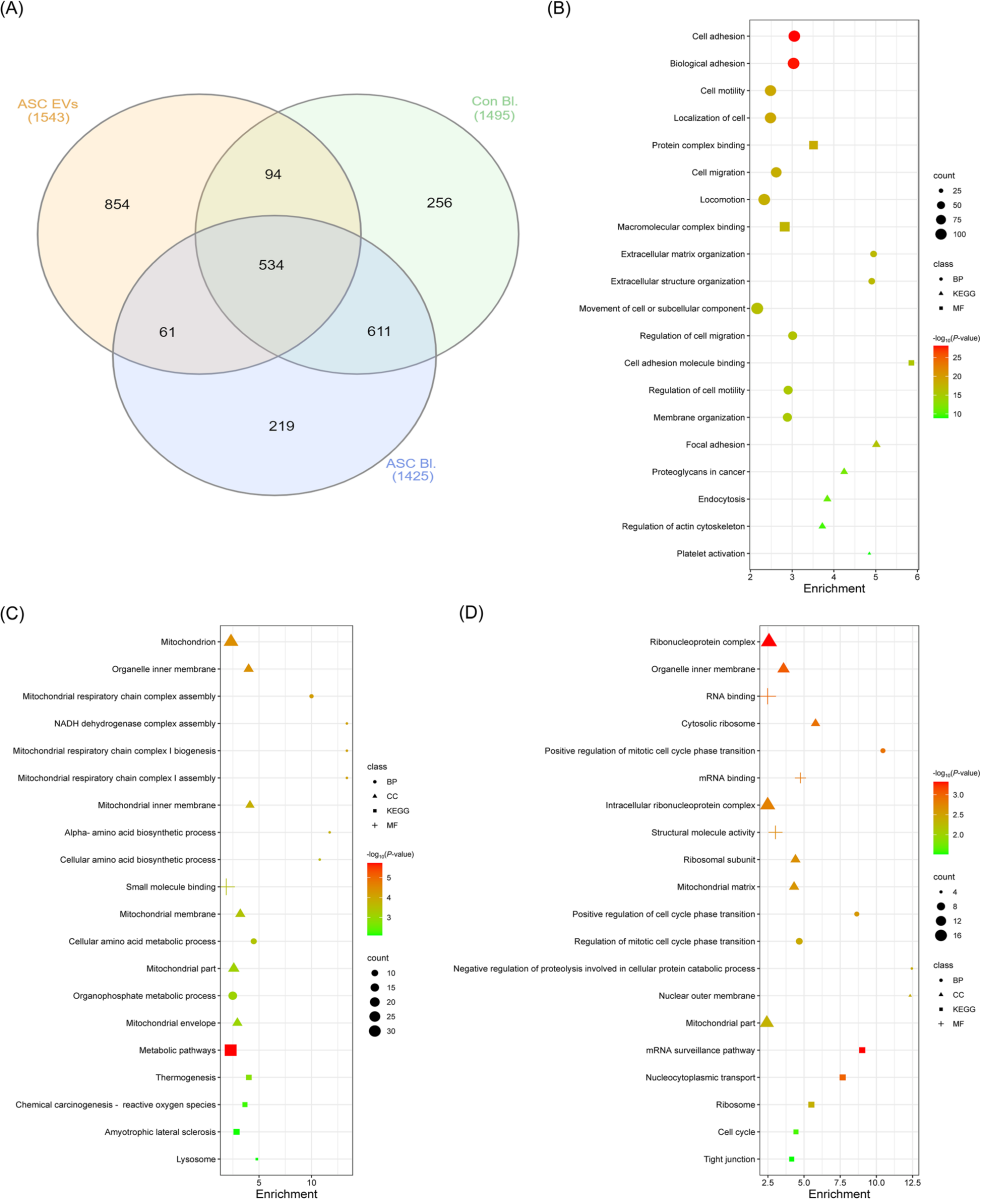
**A** Ven-diagram of proteomics analysis. Gene Ontology (GO) terms were shown for functional analysis including biological process (BP), cellular component (CC), molecular function (MF), and Kyoto Encyclopedia of Genes and Genomes (KEGG) analysis for pathway. **B** GO-terms for ASC-EVs. **C** GO-terms for Con Bl. **D** GO-terms for ASCs Bl. ASC, adipose-derived stem cells

Proteomic analysis was performed to analyze the protein content of ASC-EVs, control group blastocysts, and ASC-EV-supplemented group blastocysts. Results demonstrated that 1,425, 1,495, and 1,543 proteins were detected in the ASC-EVs, control group blastocysts, and ASC-EV-supplemented group blastocysts, respectively (Fig. 4A). Functional analysis revealed genes in ASC-EVs that were highly enriched in extracellular matrix organization (GO:0030198) and extracellular structure organization (GO:0043062) in various BP; highly enriched KEGG, including focal adhesion and proteoglycans in cancer and highly enriched MF, including cell adhesion molecule binding (Fig. 4B). Functional analysis of the control group blastocysts revealed the gene expression associated with high enrichment in BP, including mitochondrial respiratory chain complex assembly, NADH dehydrogenase complex assembly, mitochondrial respiratory chain complex I biogenesis, alpha-amino acid biosynthetic processes, and cellular amino acid biosynthetic processes (Fig. 4C). Functional analysis of the ACS-EV-supplemented group blastocysts showed gene expression associated with the high enrichment in BP, including negative regulation of proteolysis involved in cellular protein catabolic process, positive regulation of mitotic cell cycle phase transition, and positive regulation of cell cycle phase transition. Moreover, blastocysts supplemented with ACS-EVs showed the expression of highly enriched CC, including the nuclear outer membrane, and highly enriched KEGG, including the mRNA surveillance pathway and nucleocytoplasmic transport (Fig. 4D).

#### Gene Ontology

**Fig. 5:**
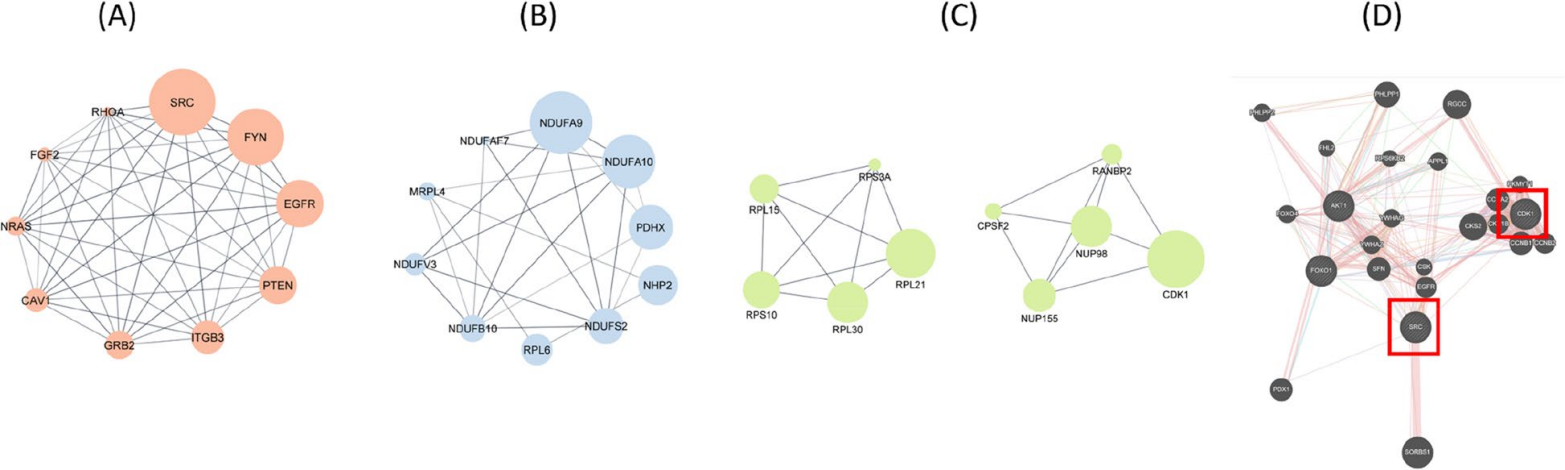
The top ten Hub genes were identified based on their degree of connectivity. The bigger the node, the higher the degree of connectivity. **A** ASC-EVs. **B** Con Blastocyst. **C** ASC-EVs treated Blastocyst. **D** GeneMANIA terms show gene interaction between SRC and CDK1. ASC, adipose-derived stem cells; EVs, extracellular vesicles; SRC, tyrosine-protein kinase; CDK1, Cyclin-dependent kinase 1

The top 10 hub genes were identified based on the degree of connectivity in the ASC-EVs, control group blastocysts, and ASC-EV-supplemented group blastocysts (Fig. 5A–C). To identify the connections between ASC-EVs and blastocysts derived from the ASC-EV-supplemented group, we used GeneMANIA to confirm the connectivity between the highly influential *SRC* in ASC-EVs and the highly influential *CDK1* in the ASC-EV-supplemented group blastocysts from the 10 hub genes detected (Fig. 5D). *SRC* and *CDK1* impacted the regulation of cell division and proliferation.

### The effect of SRC protein derived from ASC-EVs on porcine embryo development

#### Embryonic development

We divided the groups using SRC Inh and examined the embryo development rates to investigate the role of SRC influx through ASC-EV supplementation in early porcine embryo development. The groups were designated as control, SRC Inh, SRC Inh + ASC-EVs, and ASC-EVs. The experiment was performed 6 times independently, with 194 embryos cultured per group. While comparing cleavage rates, the SRC Inh group exhibited a significant decrease in cleavage rate than other groups. Furthermore, in the SRC Inh + ASC-EV group, there was a significant increase in the cleavage rate compared with that in the SRC Inh group, indicating a recovery trend. However, the cleavage rate in the SRC Inh + ASC-EV group was significantly lower than that in the control and ASC-EV-supplemented groups (Table 2). The cleavage rate in the ASC-EV-supplemented group also showed a numerical increase compared with that in the control group, but the difference was not significant.

**Table 2:** Effect of SRC introduced through ASCs-derived EVs in preimplantation porcine parthenogenetic embryo development

Groups	Number of embryos		
	**Cultured**	**Cleaved (%)**	**Developed from Bl. (%)**
Control	194	146 (75.3 ± 1.2)^a^	47 (24.2 ± 0.7)^b^
SRC Inh	194	112 (57.6 ± 1.6)^c^	26 (13.5 ± 1.2)^d^
SRC Inh + ASC-EVs	194	127 (65.5 ± 1.1)^b^	35 (18.1 ± 1.3)^c^
ASC-EVs	194	154 (79.4 ± 1.9)^a^	56 (28.9 ± 0.9)^a^

^a−d^Within a column, values with different superscript letters vary significantly among the four groups (*P* < 0.05, *n* = 6)

*ASC-EVs *extracellular vesicles derived from adipose-derived stem cells, *SRC Inh *Tyrosine-protein kinase inhibitor group, *Bl. *Blastocyst

However, blastocyst formation rates showed significant differences among all groups, with the ASC-EV-supplemented group exhibiting the highest blastocyst formation rate. This demonstrates that adding ASC-EVs derived SRC significantly influences the enhancement of blastocyst formation rates in porcine (cleaved rate: 75.3% ± 1.2%, 57.6% ± 1.6%, 65.5% ± 1.1% and 79.4% ± 1.9%; Blastocyst formation rate: 24.2% ± 0.7%, 13.5% ± 1.2%, 18.1% ± 1.3% and 28.9% ± 0.9% for control, SRC Inh, SRC Inh + ASC-EVs, and ASC-EVs groups, respectively, Table 2).

#### Role of ASC-EV-derived SRC in modulating downstream pathways during early porcine embryonic development

We investigated the influence of SRC on porcine embryo development. We quantitatively evaluated the concentrations of SRC, AKT, and phosphorylated AKT (pAKT) in embryos using Western blotting to examine the impact of SRC on AKT activation at the protein level. Quantification was performed by normalizing to β-actin. The signal intensity of the SRC protein was low in the control and SRC Inh groups, and the control and SRC Inh groups showed no significant difference. Furthermore, in the SRC Inh + ASC-EV group, the signal intensity of the SRC protein was upregulated.

**Fig. 6:**
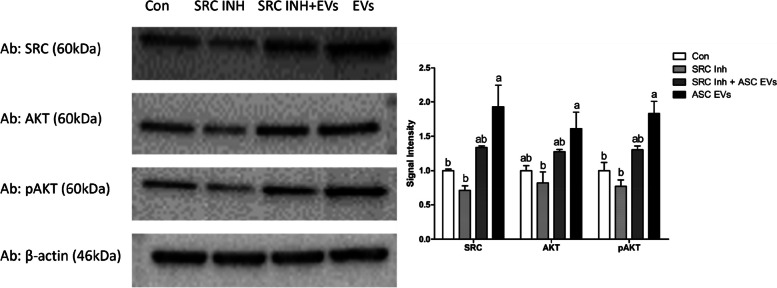
Western blot membrane images and quantitative comparison of proteins regarding the influence of SRC influx through ASC-EVs on the AKT pathway in embryos (*n* = 3). ASC, adipose-derived stem cells; EVs, extracellular vesicles; SRC, tyrosine-protein kinase; AKT, protein kinase B. ^a,b^Bars without a common letter are significantly different (*P* < 0.05)

However, in the ASC-EV group, a significantly higher signal intensity of the SRC protein was observed compared with that in the other groups. The detection of AKT was the lowest in the SRC Inh group, with a significant increase observed in the ASC-EV-supplemented group. The control, SRC Inh + ASC-EV, and ASC-EV groups exhibited an increasing trend in AKT expression, although no significant differences were observed. The signal intensity of pAKT showed similar results as SRC protein, showing low signals in the control and SRC Inh groups, a numerical increase in the SRC Inh + ASC-EVs group, and a significantly higher signal intensity in the ASC-EV-supplemented group. These results indicate that adding ASC-EVs to the existing embryo development system increases blastocyst formation rates in porcine embryos and enhances embryo quality by upregulating pAKT during the early embryo stage at the 2- to 4-cell stage (Fig. 6).

## Discussion

Our results demonstrated that supplementing the IVP embryo system with ASC-EVs improves the developmental competence of porcine embryos. Supplementation with ASC-EVs significantly increased blastocyst production efficiency by reducing OS and improving mitochondrial activity during the early embryonic stage. Furthermore, the rate of apoptotic cells in the blastocyst stage decreased, and the total number of cells increased in the ASC-EV-supplemented group.

To investigate whether the isolated ASC-EVs had any effect on embryonic development, we supplemented the culture medium with ASC-EVs following parthenogenetic activation and observed embryo development. The results demonstrated a non-significant difference in the cleavage rate between the ASC-EV-supplemented and control groups. However, the rate of blastocyst formation was significantly higher in the ASC-EV-supplemented group. Therefore, we speculated that ASC-EVs improved embryo quality and sequentially measured intracellular GSH and ROS levels and mitochondrial activity in the early embryonic stage. Unlike the cleavage rate, the intracellular GSH and ROS levels in the 2- to 4-cell stage embryo showed significant differences among the groups, reflecting the antioxidative nature of ASC-EVs. Similar findings have been reported for mesenchymal SC-derived EVs, which play an antioxidant role by delivering proteins and micro RNAs to cells [50].

Mitochondrial activity is closely associated with cellular OS and is correlated with ROS levels [51]. OS refers to an imbalance between ROS production and neutralization by antioxidants. These high levels of ROS cause mitochondrial dysfunction, which further contributes to generating intracellular ROS and inducing a vicious cycle that promotes OS [52, 53]. Our results showed that the mitochondrial activity of early embryos produced in the ASC-EV-supplemented group was higher than that produced in the control group. Considering the previous GSH and ROS levels and mitochondrial activity measurement results, we assumed that ASC-EVs delivered molecules with specific biological functions. These ASC-EV-delivered molecules may regulate OS and mitochondrial activity in the early embryo, reducing the rate of apoptosis.

ROS-induced OS causes DNA damage in embryos, leading to cellular apoptosis [54]. Supplementation of the culture medium with ASC-EVs led to reduced OS and mitochondrial activation during early porcine embryo development. Therefore, porcine embryos cultured in the ASC-EV-supplemented medium showed a significantly higher blastocyst formation rate than the control group. ASC-EV supplementation resulted in the production of high-quality blastocysts. The TUNEL assay was performed to determine whether ASC-EV supplementation affected cellular apoptosis during in vitro embryonic development. The results demonstrated that the proportion of apoptotic cells with damaged DNA was significantly reduced in the ASC-EV-supplemented group blastocysts compared with those in the control group. Additionally, in the ASC-EV-supplemented group, the total cell count in the blastocysts was significantly higher than that in the control group.

The positive effect of ASC-EV supplementation on embryonic development was supported by the improved expression levels of pluripotency- and apoptosis-related genes. The results demonstrated an increased expression of *OCT4, NANOG*, and *SALL4* in the blastocysts produced in the ASC-EV-supplemented group compared with those in the control group. Furthermore, the expression of *BAX*, a pro-apoptosis factor, significantly decreased in the ASC-EV-supplemented group, but the expression of the anti-apoptotic gene *BCL2* showed no variation among the groups. These findings provide genetic evidence that complements the decrease in apoptotic cells observed in the TUNEL assay.

Based on these outcomes following ASC-EV supplementation, we recognized the need to further explore the constituents of ASC-EVs and ascertain which proteins are conveyed, leading to improvements in embryonic quality and augmented blastocyst cell counts. Consequently, a proteomic analysis of proteins encapsulated within ASC-EVs was performed, and alterations in porcine blastocyst proteins were evaluated based on ASC-EV supplementation. Proteomic analysis revealed 1,425, 1,495, and 1,543 proteins in ASC-EVs, control group blastocysts, and ASC-EV-supplemented group blastocysts, respectively. Analysis of the biological functions of the detected proteins using the GO terms showed that the enrichment of proteins associated with cell adhesion molecule binding was high in ASC-EVs, and that which is positive in mitosis was found in the blastocysts of the ASC-EV-supplemented group. Considering the correlation between the increased total cell number in blastocysts observed in the TUNEL assay and the results of protein detection, we believe that the protein supplied through ASC-EVs positively affects cell division in porcine embryos. In addition, the top ten hub genes with functional relevance were identified. SRC and CDK1, which are closely associated with the cell cycle, were highly expressed in ASC-EVs and ASC-EV-supplemented blastocysts, respectively.

SRC Family Kinases (SFKs) are primary mediators of external signaling in cells. Their activation influences cell–cell interactions, mobility, structural changes, differentiation, and division, potentially regulating cell positioning, movement, and differentiation during embryonic development [55]. Therefore, we focused on elucidating the role of SRC in cell division and embryonic development. To confirm the transfer of SRC, parthenogenetic embryos were first treated with an SRC inhibitor to inhibit the tyrosine kinases of SRC. ASC-EVs were then added to the medium to check the delivery of SRC to the embryos. ASC-EV supplementation resulted in an increased number of porcine embryos, reflected by higher SRC expression and increased CDK1 levels, indicating the importance of SRC in cell division and embryonic development. We hypothesized that improved CDK1 expression could be transferred to porcine embryos, resulting in improved embryonic development by regulating cell division.

Additionally, SRC delivered through ASC-EV supplementation affects the activity of Akt, which inhibits the FOXO1 protein and promotes cell division [56, 57]. FOXO1 is inhibited by SRC-induced AKT phosphorylation, which activates pAKT and inhibits FOXO1. In addition to CDK1, Cyclin B is upregulated, which induces cell proliferation. These findings provide valuable insights into how ASC-EVs contribute to the increased cell numbers in porcine.

In our study, we were unable to investigate the organic substances that could be implicated in the biological functions of ASC-EV cargo, including genetic materials, proteins, lipids, intracellular vesicles, and EVs known as exosomes. However, future research holds promise for additional insights into EV-derived cargo through advanced studies that can regulate the biological pathways of EV-receiving cells and potentially facilitate their application. Furthermore, the advancement of techniques for producing and delivering EVs synthetically and the introduction of the desired cargo could create new avenues for applications in various biomedical fields, such as gene delivery, cell differentiation induction, and disease treatment.

## Conclusion

In conclusion, the changes observed in blastocysts produced in the group supplemented with ASC-EVs positively impact the early development of IVP porcine embryos, leading to increased blastocyst formation rates and reduced cellular apoptosis. These results demonstrate that ASC-EVs positively affect porcine embryo quality, particularly by increasing the expression of genes associated with pluripotency and reducing apoptosis. The results of the TUNEL assay and gene expression analysis supported this. In addition, the delivery of SRC proteins significantly increased the pAKT/AKT ratio, facilitating cell division and consequently augmenting the total number of porcine blastocysts. This elucidates the potential of EV supplementation as a novel approach to enhance the efficiency of IVP embryo cloning.

## Supplementary Information

**Aditional file 1: Table S1** List of media components and antibodies for ASC differentiation and characterization. **Table S2** List of primers for qPCR analysis of parthenogenetic embryos. **Table S3** List of antibodies used to analyze the effects of SRC on embryonic development.

## Data Availability

All data generated or analysed during this study are included in this published article and its supplementary information files.

## Abbreviations

AKT: Protein kinase B
ASC: Adipose derived stem cell
BP: Biological process
CC: Cellular component
CDK1: Cyclin-dependent kinase 1
DAVID: Database for Annotation, Visualization, and Integrated Discovery
EV: Extracellular vesicle
GO: Gene Ontology
GSH: Glutathione
H2DCFDA: 2′,7′-Dichlorodihydrofluorescein diacetate
IVP: In vitro production
KEGG: Kyoto Encyclopedia of Genes and Genomes
KlF4: Kruppel-like factor 4
MF: Molecular function
OCT4: Octamer-binding transcription factor 4
PBS: Phosphate-buffered saline
ROS: Reactive oxygen species
SC: Stem cell
Sox2: Sex-determining region Y (SRY)-box 2
SRC: Tyrosine-protein kinase
TEM: Transmission electron microscopy
TUNEL: Terminal deoxynucleotidyl transferase dUTP nick end labeling
