# Correction: Embryotrophic effect of exogenous protein contained adipose-derived stem cell extracellular vesicles

**DOI:** 10.1186/s40104-024-01127-z

**Published:** 2024-11-29

**Authors:** Seonggyu Bang, Ahmad Yar Qamar, Sung Ho Yun, Na‑Yeon Gu, Heyyoung Kim, Ayeong Han, Heejae Kang, Hye Sun Park, Seung II Kim, Islam M. Saadeldin, Sanghoon Lee, Jongki Cho

**Affiliations:** 1 College of Veterinary Medicine and Research Institute for Veterinary Science, Seoul National University, Seoul, 08826 Republic of Korea; 2 College of Veterinary Medicine, Chungnam National University, Daejeon, 34134 Republic of Korea; 3 College of Veterinary and Animal Sciences, Jhang Sub-Campus of University of Veterinary and Animal Sciences, Lahore, 54000 Pakistan; 4 Korea Basic Science Institute (KBSI), Ochang, Chungcheongbuk-Do 28119 Republic of Korea; 5 Viral Disease Research Division, Animal and Plant Quarantine Agency, Gimcheon, Gyeongsangbuk-Do 39660 Republic of Korea; 6 Department of Plastic and Reconstructive Surgery, Vascularized Composite Allotransplantation (VCA) Laboratory, Johns Hopkins School of Medicine, Baltimore, MD 21205 USA; 7 Comparative Medicine Department, King Faisal Specialist Hospital & Research Centre, Riyadh, 11211 Saudi Arabia

**Correction** **:** **J Animal Sci Biotechnol 15, 145 (2024)**

**https://doi.org/10.1186/s40104-024-01106-4**

Following publication of the original article [1], the authors reported an error in authors’ affiliations due to typesetting error, where the 3^rd^ and 4^th^ institutions are the same. Also, a full stop was erroneously added to author Seung II Kim’s name.

The full list of authors and affiliations is changed from:

Seonggyu Bang^1,2^, Ahmad Yar Qamar^3^, Sung Ho Yun^5^, Na-Yeon Gu^6^, Heyyoung Kim^2,7^, Ayeong Han^1,2^, Heejae Kang^1,2^, Hye Sun Park^4^, Seung II. Kim^4^, Islam M. Saadeldin^2,8^, Sanghoon Lee^2^ and Jongki Cho^1^^*^

1 College of Veterinary Medicine and Research Institute for Veterinary Science, Seoul National University, Seoul 08826, Republic of Korea.

2 College of Veterinary Medicine, Chungnam National University, Daejeon 34134, Republic of Korea.

3 College of Veterinary and Animal Sciences, Jhang Sub-campus of University of Veterinary and Animal Sciences, Lahore, Pakistan.

4 College of Veterinary and Animal Sciences, Jhang, Sub-Campus of University of Veterinary and Animal Sciences, Lahore 54000, Pakistan.

5 Korea Basic Science Institute (KBSI), Ochang, Chungcheongbuk‑Do 28119, Republic of Korea.

6 Viral Disease Research Division, Animal and Plant Quarantine Agency, Gimcheon, Gyeongsangbuk‑Do 39660, Republic of Korea.

7 Department of Plastic and Reconstructive Surgery, Vascularized Composite Allotransplantation (VCA)

Laboratory, Johns Hopkins School of Medicine, Baltimore, MD 21205, USA.

8 Comparative Medicine Department, King Faisal Specialist Hospital & Research Centre, Riyadh 11211, Saudi Arabia.

To:

Seonggyu Bang^1,2^, Ahmad Yar Qamar^3^, Sung Ho Yun^4^, Na‑Yeon Gu^5^, Heyyoung Kim^2,6^, Ayeong Han^1,2^, Heejae Kang^1,2^, Hye Sun Park^4^, Seung II Kim^4^, Islam M. Saadeldin^2,7^, Sanghoon Lee^2^ and Jongki Cho^1^^*^

1 College of Veterinary Medicine and Research Institute for Veterinary Science, Seoul National University, Seoul 08826, Republic of Korea.

2 College of Veterinary Medicine, Chungnam National University, Daejeon 34134, Republic of Korea.

3 College of Veterinary and Animal Sciences, Jhang Sub-campus of University of Veterinary and Animal Sciences, Lahore 54000, Pakistan.

4 Korea Basic Science Institute (KBSI), Ochang, Chungcheongbuk‑Do 28119, Republic of Korea.

5 Viral Disease Research Division, Animal and Plant Quarantine Agency, Gimcheon, Gyeongsangbuk‑Do 39660, Republic of Korea.

6 Department of Plastic and Reconstructive Surgery, Vascularized Composite Allotransplantation (VCA)

Laboratory, Johns Hopkins School of Medicine, Baltimore, MD 21205, USA.

7 Comparative Medicine Department, King Faisal Specialist Hospital & Research Centre, Riyadh 11211, Saudi Arabia.

The original article [1] has been updated.
