# Supplementary material for: Embryotrophic effect of exogenous protein contained adipose-derived stem cell extracellular vesicles
Source: J Anim Sci Biotechnol. 2024 Nov 3;15:145. doi: 10.1186/s40104-024-01106-4 (PMC11531693; doi:10.1186/s40104-024-01106-4)
Supplement: Supplementary file 1 — Aditional file 1: Table S1 List of media components and antibodies for ASC differentiation and characterization. Table S2 List of primers for qPCR analysis of parthenogenetic embryos. Table S3 List of antibodies used to analyze the effects of SRC on embryonic development. [file 40104_2024_1106_MOESM1_ESM.docx]

**Additional file 1**

**Table S1** List of media components and antibodies for ASC differentiation and characterization

| **Differentiation medium** | | | | | | | | | |
| --- | --- | --- | --- | --- | --- | --- | --- | --- | --- |
|  | **Medium** | **Dexamethason, μmol/L** | **Indomethacin, μmol/L** | | **3-Isobutyl-1-methylxanthine,**  **μmol/L** | **Insulin, μg/mL** | **Β-glycerophosphate, mmol/L** | **L-ascorbic acid 2-phosphate, μmol/L** | **TGF-β1, ng/mL** |
| Adipogenic | DMEM-LG | 1 | 200 | | 500 | 0.5 | - | - | - |
| Chondrogenic | DMEM-LG | 100 | - | | - | - | - | 50 | 10 |
| Osteogenic | DMEM-LG | 100 | - | | - | - | 10 | 50 | - |
| **List of antibodies (Ab)** | | | | | | | | | |
| **Ab** | **Name** | | | **Company** | | | **Isotype** | | |
| CD29 | Mouse anti-Pig CD29 | | | BD Biosciences,  552369 | | | Mouse IgG1 | | |
| CD34 | Rabit monoclonal anti-CD34 | | | Abcam, ab81289 | | | Rabbit IgG | | |
| CD44 | FITC Rat anti-Mouse CD44 | | | BD Biosciences,  553133 | | | Rat IgG2b | | |
| CD90 | PE Mouse anti-Human CD90 | | | BD Biosciences,  555596 | | | Mouse IgG1 | | |

**Table S2** List of primers for qPCR analysis of parthenogenetic embryos

| **Gene** | | **Primer sequences (5’-3’)** | | **Size, bp** | **Accession No.** |
| --- | --- | --- | --- | --- | --- |
|  |  | **Forward** | **Reverse** |  |  |
| House keeping | *GAPDH* | AGAAGGTGGTGAAGCAGG | AGCTTGACGAAGTGGTCG | 154 | XM_003126531 |
| Apoptosis | BAX | ACTTCCTTCGAGATCGGC | GGCCACGAAGATGGTCAC | 110 | XM_003127290 |
|  | BCL2 | TTCTCTCGTCGCTACCGC | CCAGTTCACCCCATCCCT | 123 | XM_021099593 |
| Pluripotent | *OCT4* | GAAGGTGTTCAGCCAAACGAC | CGATACTTGTCCGCTTTC | 185 | NM_001113060.1 |
|  | *NANOG* | AGCCTCCAGCAGATGCAAGAACTCT | TTCTGCCACCTCTTACATTTCATTCG | 181 | FJ882402.1 |
|  | *SALL4* | ATCCACCTCCGCTCCCATACC | CGTTGCCTGCCGTCATCTTGT | 166 | NM_001114673.1 |

**Table S3** List of antibodies used to analyze the effects of SRC on embryonic development

| **Ab** | **Name** | **Company** |
| --- | --- | --- |
| SRC | SRC Monoclonal Antibody | Invitrogen, AHO1152 |
| AKT | Akt Antibody | Cell signaling technology, 9272 |
| pAKT | Phospho-AKT (Ser473) Antibody | Cell signaling technology, 9271 |
| Β-actin | Monoclonal IgG1 k beta actin | Santacruz, sc-47778 |
